# Operative Dentistry

**Published:** 1876-10

**Authors:** Sam’l E. Goe


### OPERATIVE DENTISTRY.

BY SAM'L E. GOE, D. D. S.

*Read before the California State Dental Association, June 13, 1876.*

Mr. President and Gentlemen:—When apprised of my appointment as one of the committee on operative dentistry, I felt that my duties as a member of that committee would be light; that an abler pen than mine would note the progress this branch of our speciality had made during the last twelve months; still I resolved to show at least a willingness to requite the respect shown me by the appointment, deciding that whatever I might write should be presented as a sort of supplementary essay or addenda, to the report proper. However, should I deviate from the true purpose of the committee I hope the digression will be pardoned, on the ground that what I offer is not to be considered *the* report.

What we understand by operative dentistry, embraces by far the greater portion of the dentist's duties, thoughts and ambition; and upon his ability to preserve teeth by filling he builds his reputation as a dental practitioner. At least the public always attributes the failure of any conservative dental operation to the lack of mechanical skill in its performance; taking cognizance of no concomitant destructive agency which may have been its direct cause. In fact, it is very common for operators themselves to pass carelessly over conditions of the mouth which may work adversely to all their efforts. Sex, age, temperament, the structure of the teeth operated upon, local and other causes are too often lost sight of entirely, and the whole attention of the operator directed toward arranging, mechanically, in cavities, small pellets of gold in the best and neatest manner. I do not wish to be understood as undervaluing any achievement in the mechanical perfections of dental operations; on the contrary, I regard such ambition as worthy and laudable, and absolute thoroughness indispensably neccessary to the preservation of teeth operated upon, even in the most favorable cases; but this thoroughness can not, or should not, dispense with considering the conditions mentioned, nor with the effort to correct any evil arising from them.

In no instance is this carelessness exhibited, as in the treatment intended as conservative of exposed pulps. This operation is often performed properly so far as the mechanical introduction of the material is concerned, either for the capping or the plugging of the cavity, yet it is done in the face of certain conditions which render success almost impossible.

In regard to the excavation of cavaties, so much has been said, so many directions given as "how to cut," "when to stop," etc., etc., that one would think we must have reached the ultimatum. But notwithstanding our rigid course of drilling on this point, we are called to witness almost daily, failures which may justly be attributed to careless excavation, or ignorant shaping of cavities before introducing the plug. We can have no formula or prescribed set of rules sufficient to govern us in the preparation of cavities. In this, as in other particulars our judgment must guide us.

The structure and density of the teeth, the particular tooth and the manner of its occlusion with its fellows must all be taken into account.

However, the young operator who resolves to lead a professional life, somewhat higher than that of vulgar accident, may do well to put himself under some system of rules. or precepts. Through rigid adherence to them, he may, to a certain degree, ensure success to his efforts; but when experience shall have ripened his judgment, these rules, manuals, codes of mechanical drill and systems of restrictions may be discarded.

A deeper and more intelligent order of practice will take authority over him, and so far as mastery over mechanical laws are concerned, render him in turn master of the situation.

One rule in excavating may be established, and for that matter may be adhered to through life: always cut clean, smooth borders, and when the walls of the cavity are parallel, terminating at the margins by a sharp angle, this should be rounded off, making what is known in mechanics, as a *countersink.* This will render perfect the impaction of the gold, with out danger of chipping the edges of the cavity, either with the plugging instrument, or in mastication afterwards.

The second rule should be, to free the cavity so far as possible from under cuts; and running counter to this last is another, forbidding the cutting away of the tooth in a manner to weaken it. Between these two opposing rules judgment must decide. In simple cases every portion of disorganized dentine or tooth substance should be removed; though when the thorough removal of this would expose the pulp, a portion of partially decalcified dentine may remain without any injurious result. Leaving this subject somewhat abruptly, I will pass to the consideration of another.

Since the introduction of Dr. Arthur's little book, now become so universally known to the profession, it has been the practice with many to use the file freely in making permanent separations between the teeth. Without denying that the author's method has some value, I would advise judgment and moderation in the use of the file or disk; or, in fact, any means employed by which the beauty and usefulness of the dental organs and the consequent comfort of the patient may be destroyed.

It is claimed that when free separation is made between the teeth, especially when proximate cavities are to be treated by filling, cleanliness is secured beyond a peradventure, that these parts are self-cleansing, by virtue of their separation, and kept free from injurious agents by natural causes. I can not wholly endorse this theory. A dentist's mission is not only to save teeth, but as far as possible to keep them in their normal condition, and restore their beauty and usefulness when impaired or lost. While I do not deny that teeth may be filed away to considerable extent without danger of being attacked by caries, still I think every one in his own experience, if he will look into it, will find instances wherein decay has made its appearance upon the surfaces of teeth, upon which the file has been freely used, perhaps, with the object of warding off this attack. Separation will not always secure immunity from decay, and the more we study the local and general conditions of the tissues, we deal with their tendency to disease or destructability, and the rationale of any proposed course of treatment, the better we shall be able to cope with the influences which work their ruin. It is not judicious but indiscriminate filing which I condemn.

In treatment by filling of teeth which have lost any portion of their original shape, it should be your study to know how nearly we can restore their form and usefulness, and still ward off the attack made by destructive agents, and without anticipating trouble, endeavor to teach prophylactic treatment to those who seek our care.

I approach the subject of exposed pulps with no small amount of timidity, yet I can not ignore it altogether. Notwithstanding the wonderful progress that has been made in successfully treating pulps after exposure by caries, this subject still needs better to be understood. The advantages of keeping alive this little organ, and of retaining its functions, are too apparent to require stating here.

The physiology and pathology of the dental pulp probably belong to another department, and should be left out of a treatise on operative dentistry, but the conditions are always to be considered, before instituting any operative treatment, which must in every case, be dependent upon them, for its mode of action, hence, this subject can not be treated in sections, with any degree of satisfaction, either to the essayist or to others.

In the majority of cases of exposure presented to us the pulp is already in a pathological condition, a state of inflammation to some degree, and in the hands of the intelligent operator, the treatment will depend upon the extent of this inflammation.

Prof, Taft in his work on operative dentistry, enumerates the considerations to be taken into account as follows:

First, the constitution of the system.

Second, the condition of the mouth and teeth.

Third, the condition of the pulp.

Fourth, the size of the orifice at which it is exposed.

Fifth, whether it is of recent or remote origin.

Sixth, if in a tooth of more than one root,

Seventh, the location of the tooth in the mouth, and the cavity in the tooth.

These considerations seem to have been enumerated in the order of their relative importance; for surely the first inquiry in taking hold of a case should be, what can we reasonably expect from the general health of the system? And just in proportion as we are able to solve this problem, shall we be able to cope with the contingencies against us in the treatment of exposure of the dental pulp. Without first taking steps to restore the general health, how utterly foolish, in cases of long standing, to attempt the operation for capping an exposed pulp. For a patient with a low grade of vital energy, an inflammatory diathesis, in fact, when no assistance can reasonably be expected from nature in resisting irritation caused by the introduction of foreign substances directly in contact with so delicate a tissue.

Probably the condition of the pulp should be the next consideration. Its tone, the condition of the mouth and teeth, the size of the orifice of exposure, whether of recent or remote origin, etc., are all important questions, and worthy of notice. Yet the vital energy of the system and the condition of the pulp, are, in my mind, the two great questions to be considered.

In many instances, the condition of the pulp, will admit of immediate operation; capping, and the subsequent filling of the cavity may be done at once, no therapeutic treatment being called for. But, unfortunately, some, I might say the majority of cases, are not in this state; frequently, our first ministration is to relieve the intense pain caused by an already advanced stage of inflammatory action. These are the more complicated cases, with which we have to deal, calling forth the keenest exercise of judgment and skill; again, in cases of remote origin, the pulp will be found to be in a state of chronic inflammation, requiring quite different modes of treatment.

When topical therapeutic treatment is called for, an accurate diagnosis should be made, as the means to be employed will depend upon this; we must arrive at the means to be used by a course of symptomatic reasoning, and our judgment must decide, as to the proper time for capping and filling. In the mild forms of diseased action, mild remedies should be used, bringing heroic treatment into play, only when there is urgent need of it; this assertion, naturally involves the propriety of always using escharotics, such as carbolic acid, and kindred agents, which in my mind is not intelligent practice. There is as much propriety in administering a dose of squills for an ingrowing nail, as in the application of carbolic acid or creosote in every case of diseased action; sending the patient away for a day or two, expecting that he will return with the pulp in the proper condition for operating.

In cases of slight inflammatory action escharotics should never be used, there being no demand for them. The application of some anodyne after carefully excavating the cavity would be the more reasonable course to pursue.

Various materials and methods for capping exposed pulps have been and are still used, almost every practitioner has some method which in his hands is more successful than would be the adoption in toto of any one's else. Still as all aim at the same result, improvement in minor points may be made by the free interchange of methods and experience. My method in general is to open well the cavity of decay until ready access to every portion of it is made when the carious tooth substance is carefuly removed. Sometimes a thin layer of partially decalcified dentine may be left over the pulp, This however is determined by the character of the decay. Afterward, the cavity is finally prepared for the reception of the filling. If the pulp has been fully exposed, and no therapeutic treatment required, it is covered with a thin coating of liquid gutta percha or collodion. As the material for capping, generally is oxy-chloride of zinc, a small portion of the oxide is mixed, *sometimes* with a little creosote, to a pasty consistency, and sufficient of this mixture introduced to afford protection to the pulp; dry this off with a piece of spunk or absorbent paper, and cover with a layer of oxy-chloride. This, when properly set, will afford a protection to the pulp, both from pressure, and thermal changes caused by the conducting properties of the metallic filling. If thought best to fill permanently at the same sitting, sufficient time is allowed for the thorough hardening of the capping; if not, the cavity can be left temporarily stopped with the oxy-chloride, and the operation completed at some future time.

It is not alone, in cases wherein the pulp is actually exposed, that the process of capping is required. Oftentimes, when excavating teeth, we discover that caries has almost invaded the sanctum of this little organ, leaving only a thin covering of dentine over it; if the covering be very slight, and the cavity is to be filled with a metallic substance, it matters not if what does remain directly over the pulp be perfectly normal and perfectly free from disintegration, the interposition of some non-conducting material is emphatically called for. The rationale of this method must be obvious to every thinking mind.

In regard to new appliances brought to our notice during the last year I have but little to say; yet one or two things I wish to mention.

Those who were present at the last meeting of this association, will remember that Dr. S. E. Knowles presented for inspection some little corundum disks, for use in the dental engine, (since manufactured by S. S. White) for cutting out buccal and lingual fissures in the molar teeth. These are wonderful little appliances; so rapid and painless are they in their execution, that only he who has given them a trial can appreciate their value; and after learning their use, he will always be provided with them.

Another useful requisite to the operating case, is a little brush wheel which is also used in the engine. It is especially adapted to the removal of discolorations from the teeth. It kindly insinuates itself between the teeth and under the free margin of the gums, removing deposits therefrom, producing no laceration of this delicate tissue.

Mr. President, in concluding this crudely prepared paper, I wish to say that if I have given expression to a single thought that will be the means of calling from those who are older and more experienced than myself, any discussion either upon the subject matter above alluded to, or upon any thing else relating to operative dentistry, I shall feel that I have done my duty, at least all I aimed at, and I, with the rest, shall be greatly profited thereby.

Oct-2

